# Coinfection affects the phenotypic but not genetic resistance of cattle to common parasites

**DOI:** 10.1186/s12711-025-01003-y

**Published:** 2025-10-07

**Authors:** Fabio Luiz Buranelo Toral, Maria Victoria Souza, Mariana Mamedes de Moraes, Valentina Riggio, Gabriela Canabrava Gouveia, Virginia Mara Pereira Ribeiro, Osvaldo Anacleto, Eduardo Penteado Cardoso, Daniel Resende Gonçalves, Andrea Doeschl-Wilson

**Affiliations:** 1 Universidade Federal de Minas Gerais, Avenida Antonio Carlos, 6627, Belo Horizonte, MG 31270-901 Brazil; 2 The Roslin Institute and Royal (Dick) School of Veterinary Studies, University of Edinburgh, Midlothian, EH25 9RG UK; 3 Purdue University, West Lafayette, IN 47907 USA; 4 Fazenda Novo Mundo, Rod. BR 050, Km 125, Uberaba, Brazil

## Abstract

**Background:**

Genetic variation in host resistance to individual parasites is well documented in cattle; however, the influence of coinfection on these genetic responses to selection remains poorly characterized. In particular, it is unclear how concurrent exposure to multiple parasite species alters phenotypic expression, heritability estimates, or genetic correlations between resistance traits. To address these gaps, we evaluated the impact of coinfection on the genetic architecture of parasite resistance in yearling Nellore calves naturally challenged with ectoparasites (ticks) and endoparasites (gastrointestinal nematodes and *Eimeria* spp.). Using longitudinal parasite count data, we estimated genetic parameters and examined how coinfection modifies both individual parasite resistance and the genetic correlations among traits.

**Results:**

Our results confirmed that coinfection is a common phenomenon (almost ¾ of samples contained multiple parasites) and that resistance to individual parasites is a heritable trait. Furthermore, coinfection with *Eimeria* spp. reduced the phenotypic resistance to nematodes, and vice versa. We observed diverse genetic associations for resistance to different parasites, including positive, negative, and nonsignificant correlations. Notably, coinfection had no significant effect on genetic resistance to individual parasites, nor did it alter genetic variances or associations between resistance to different parasites.

**Conclusions:**

While coinfection may influence the outcomes of nongenetic parasite control programs, its impact on genetic control strategies appears minimal. In other words, genetic resistance of Nellore cattle to three key parasite species appears to be robust and unaffected by the presence of coinfection.

**Supplementary Information:**

The online version contains supplementary material available at 10.1186/s12711-025-01003-y.

## Background

Parasite infections in cattle not only lead to substantial economic losses but also compromise host health and fitness. In Brazil, ecto- and endoparasites such as ticks and gastrointestinal nematodes (GIN) are major contributors to the financial burden on the beef cattle industry, accounting for estimated annual losses of US$7.11 billion and US$3.24 billion, respectively [1, 2]. From an animal welfare perspective, these parasites damage the skin and hide [3], act as vectors of various diseases [4] and reduce average daily gain in calves [5]. Similarly, *Eimeria* spp. (EIM), a genus of protozoan that cause coccidiosis, poses a significant threat to cattle health. Clinical manifestations such as diarrhea, dehydration, and reduced growth performance can occur [6], with economic losses of approximately $723 million annually [7–9].

Coinfection—the simultaneous infection of a host by multiple parasite—is commonly observed in cattle, with ticks, GIN and EIM frequently infecting animals concurrently [5, 10–12]. Such coinfections can alter the dynamics and prevalence of individual parasite infections through inter-parasite or parasite-host interactions. These interactions may influence disease severity [10, 13, 14], compromise the efficacy of targeted chemical treatments [15], and contribute to the development of anthelmintic or acaricide resistance [16–18]. Consequently, coinfection presents a major challenge to effective parasite control strategies and animal health worldwide.

Selective breeding for host resistance has emerged as a suitable alternative to mitigate the effects of parasite infections and address the rising issue of drug resistance [18–20]. However, most genetic studies on parasite resistance in cattle have focused on single-parasite models [11, 21–23]. Given the ubiquitous nature of coinfection, research considering multiple parasites within the same host has received surprisingly little attention [12, 24, 25]. Furthermore, estimates of genetic parameters for resistance to the same parasite species often vary across studies [11, 22, 24, 26], reflecting differences in host breed, measurement timing, statistical methodology, and potentially, coinfection status. Understanding how coinfection influences these genetic parameters is essential, as it directly impacts the design and success of breeding programs aimed at improving parasite resistance.

To date, no studies have explicitly investigated how coinfection affects estimates of genetic variance and covariance for host resistance to multiple parasites. This may be due to the logistical challenges of obtaining sufficiently detailed phenotypic and genetic data from naturally coinfected populations. Specifically, longitudinal field studies that include repeated measurements from the same individuals are rare, yet essential to disentangle the genetic bases of resistance in coinfected hosts.

The present study aims to fill this knowledge gap by leveraging a unique dataset of repeated parasite count records in Nellore cattle naturally exposed to a complex parasitic environment. We focused on one ectoparasite (cattle ticks) and two endoparasites (GIN and *Eimeria* spp.), selected for their high prevalence and well-documented impacts on cattle performance and health [2, 8, 25, 27–29]. Using these data, we investigated whether coinfection influences the host’s phenotypic and genetic resistance to individual parasites and estimated genetic correlations among resistance to different parasites.

## Methods

### Field study design and data collection

The Ethics and Animal Experimentation Committee of the Universidade Federal de Minas Gerais, Brazil (Protocol 255/2010), approved all the experimental procedures used in the present study. All animals included in this study were managed, handled, and reared according to the common standards of commercial cattle production systems in Brazil. The field study was conducted from 2011 to 2017 on a commercial farm located in the municipality of Uberaba, Minas Gerais state, Brazil (latitude 19° 24′ 33.3″ S, longitude 48° 06′ 34.5″ W, and 840 m above sea level). The region’s climate is classified as Cwa on the Köppen scale, characterized by generally hot, humid summers and cooler, relatively dry winters [30].

The population consisted of purebred Nellore male calves weaned at 240 days of age. After weaning, calves were reared in separate paddocks of *Urochloa* pasture of approximately 30 hectares, totaling eight groups per year. The calving season occurs from July to December each year. To ensure consistent grazing and parasite challenge conditions, these groups were rotated. Calves were not allowed to move from one group to another. The age of the animals used in the field study ranged from 275 to 597 days. In total, 1712 animals were included.

Calves were administered anthelmintics at birth and weaning, and sporadically in groups that were found to be more affected by GIN. No parasite counts were taken within less than 56 days after drenching. No acaricides or anticoccidial drugs were used during the study. For additional information on experimental design refer to [24].

### Parasitological investigations

The term ‘infection’ in this study refers to both infestations by cattle ticks [1, 31] and infections by GIN and EIM as determined by positive parasite counts described below. Repeated counts of the number of ticks on weaned calves were performed, and fecal samples were collected to count GIN eggs and EIM oocysts. Up to five repeated measurements per animal were taken every 56 days during the 224-day study period (days 0, 56, 112, 168, and 224) for a total of 7886 records. Tick counting was performed according to the technique of Wharton and Utech [32], in which engorging female ticks (length size > 4.5 mm) were counted on the right side of the animal. Fecal samples were collected directly from the animal’s rectum and subsequently examined according to the modified McMaster technique [33] by increasing the flotation‐solution concentration and examining a larger fecal sample per chamber. This adjustment effectively lowers the practical detection limit, improving sensitivity, while still aligning with standard McMaster performance. Specifically, the assessments of GIN and EIM infections were performed with 2 g of feces diluted in 28 mL of water. These materials were mixed and sifted, and then 2 mL of this solution was homogenized with 2 mL of a saturated flotation solution. An aliquot was taken to fill the McMaster chamber, where GIN and EIM were counted under a microscope at 10 × magnification. All counts reported in this study are the raw numbers observed in the chamber (i.e., per the 2 g of feces examined), with no additional correction factor applied. To convert these to the standard “eggs per gram” (EPG), one would multiply by 50. The GIN present were a mix of different genera, namely, *Haemonchus* (~ 60%), *Trichostrongylus* (~ 25%), *Cooperia* (~ 13%) and *Oesophagostomums* (~ 2%).

### Data editing

Out of 7886 samples, 592 samples with incomplete measurements (i.e., missing records from one or more parasites) were removed. The final data set contained 7294 records from 1712 animals. These animals were offspring of 130 bulls and 1132 cows. A recursive algorithm was used to build the numerator relationship matrix with all the known ancestors of animals with records, totaling 5,919 animals.

### Statistical analysis

We used integrated nested Laplace approximation (INLA), a nonsampling-based Bayesian inference method [34–36], implemented in R-INLA to approximate posterior marginals of the hyperparameters (variances, covariances and correlations) of the genetic animal models described below.

A priori, data were analyzed using Poisson (P) and negative binomial (NB) animal models and their zero-inflated versions (ZIP and ZINB, respectively) to assess the optimal fit for parasite count records. Model fit was assessed by the deviance information criterion (DIC) [See Additional file 1, Data Analysis], where the NB was the best model.

We performed single-trait and multiple-trait analyses that did not account for coinfection with another parasite. Single-trait analyses used a generalized linear mixed model (GLMM) with a negative binomial distribution, chosen for its optimal fit to the parasite count records in our samples. The probability mass function for the negative binomial distribution of the response ${Y}_{ij}$(parasite counts) of the $i$ th individual at time $j$ is given by:

$$f\left({Y}_{ij}={y}_{ij}|{\omega }_{i},\kappa \right)=\frac{\Gamma \left(\kappa +{y}_{ij}\right)}{\Gamma \left(\kappa \right){y}_{ij}!}{\left(\frac{{\omega }_{i}}{\kappa +{\omega }_{i}}\right)}^{{y}_{ij}}{\left(\frac{\kappa }{\kappa +{\omega }_{i}}\right)}^{\kappa },$$

Where ${\omega }_{i}$ is the mean parasite count for the $i$ th individual and $\kappa$ is the shape parameter, with expectation and variance: $E\left({Y}_{ij}|{\omega }_{i},\kappa \right)={\omega }_{i},$ $Var\left({Y}_{ij}|{\omega }_{i},\kappa \right)={\omega }_{i}+\frac{{\omega }_{i}^{2}}{\kappa }$.

As described previously, the NB model provided the best fit and was therefore used for the subsequent genetic analyses. To connect the mean count ${\omega }_{i}$ to fixed and random effects in a linear mixed modeling framework, we applied log-link function. This transformation ensures that predicted parasite counts remain positive and allows for a linear modeling framework on the logarithmic scale. The model is defined as:

$$\text{ln}\,\omega ={\varvec{X}}{{\varvec{b}}}_{\omega }+{{\varvec{Z}}}_{1}{{\varvec{a}}}_{\omega }+{{\varvec{Z}}}_{2}{{\varvec{c}}}_{\omega }+{{\varvec{Z}}}_{3}{{\varvec{g}}}_{\omega \boldsymbol{ }},$$

where, ${{\varvec{b}}}_{\omega }$ represents the fixed effect of age at measurement, while ${{\varvec{a}}}_{\omega }$**,** ${{\varvec{c}}}_{\omega }$, and ${{\varvec{g}}}_{\omega }$ denote the random effects for additive genetic, permanent environment and group-level variation, respectively. The group effect accounts for shared factors such as year-season of birth, management group, and date of measurement. The corresponding incidence matrices are ${\varvec{X}}$, ${{\varvec{Z}}}_{1}$, ${{\varvec{Z}}}_{2}$ and ${{\varvec{Z}}}_{3}$.

The vectors of random effects are assumed to follow normal distributions:

$${{\varvec{a}}}_{\omega }\left|{\varvec{A}},\right.{\sigma }_{{a}_{\omega }}^{2}\sim N\left(0,{\varvec{A}}{\sigma }_{{a}_{\omega }}^{2}\right),$$

$${{\varvec{c}}}_{{\varvec{\omega}}}\left|{{\varvec{I}}}_{{\varvec{P}}},\right.{\sigma }_{{c}_{\omega }}^{2}\sim N\left(0,{{\varvec{I}}}_{{\varvec{P}}}{\sigma }_{{c}_{\omega }}^{2}\right),$$

$${{\varvec{g}}}_{{\varvec{\omega}}}\left|{{\varvec{I}}}_{{\varvec{G}}},\right.{\sigma }_{{g}_{\omega }}^{2}\sim N\left(0,{{\varvec{I}}}_{{\varvec{G}}}{\sigma }_{{g}_{\omega }}^{2}\right),$$

where ${\varvec{A}}$ is the pedigree-based relationship matrix (5919 animals); ${\sigma }_{{a}_{\omega }}^{2}$ is the additive genetic variance; ${{\varvec{I}}}_{{\varvec{P}}}$ is the identity matrix of order $P$, the number of animals with records (1712); ${\sigma }_{{c}_{\omega }}^{2}$ is the permanent environment variance; ${{\varvec{I}}}_{{\varvec{G}}}$ is the identity matrix of order $G$, the number of groups (228); and ${\sigma }_{{g}_{\omega }}^{2}$ is the group variance. Priors for ${{\varvec{b}}}_{\omega }$ were drawn from a normal distribution, whereas those for the variances are assumed to follow inverse-Gamma distributions (0.5, 0.5).

Building on the univariate models, we extended our analysis to bivariate models using joint distributions for parameters associated with different parasite species. We conducted standard two-trait analyses for each pair of traits (*i.e.,* parasite species), excluding the effect of coinfection with a third parasite. These results, referred to as “standard,” are presented alongside those from models that account for coinfection with two or three parasites [see Additional file 1, Data Analysis].

### Assessing the impact of coinfection on host resistance

To assess the effects of coinfection on phenotypic and genetic resistance to single and multiple parasites, we categorized phenotypic records into high and low coinfection groups based on the median parasite count of the coinfecting species. Preliminary analyses using binary coinfection presence (*i.e.,* infected *vs*. not infected) resulted in highly imbalanced datasets, particularly for certain parasite combinations (*e.g.,* ticks and GIN), where most animals were coinfected. This imbalance yielded unreliable posterior marginal distributions in the Bayesian framework, justifying our decision to use a median-based threshold to create more balanced groups for analysis.

We aimed to test two core hypotheses: (i) Coinfection alters the host’s phenotypic and genetic resistance to individual parasites; (ii) Coinfection modifies the genetic correlation structure between resistance to different parasites. To test these hypotheses, we implemented a stepwise modeling strategy: (i) single-trait models with coinfection effects; (ii) two-trait models comparing low *vs* high coinfection contexts, and (iii) cross-parasite genetic correlations under coinfection.

### Single-trait models with coinfection effects

For each parasite, we assessed whether including the count of a coinfecting parasite (either as a linear covariate or a class effect, using the low/high coinfection grouping) improved model fit. This was evaluated using deviance information criterion (DIC) and whether the posterior distributions of the coinfection effect included zero [See Additional file 1].

### Two-trait models comparing low *vs* high coinfection contexts

We investigated whether genetic parameters (heritability, variance and correlations) for a given parasite changed between low and high coinfection groups. For example, tick counts under low *vs*. high GIN burden were modeled as two separate but related traits. This allowed us to estimate and compare the genetic variances and correlations, effectively treating coinfection level as a discrete environmental modifier. We conducted six GLMMs (3 parasites × 2 coinfecting species) [See Additional file 2, Table S1], and tested statistical significance via permutation test (10 replicates per parasite, random reassignment to high/low groups).

### Cross-parasite genetic correlations under coinfection

To evaluate whether genetic resistance to parasite X correlates differently with resistance to parasite Y depending on coinfection with parasite Z, we estimated cross-parasite genetic correlations within low and high coinfection strata [See Additional file 2, Table S2]. We hypothesized that genetic associations between resistance to parasites X and Y would remain consistent irrespective of the level of coinfection with parasite Z. For instance, we compared the genetic correlation between tick and *Eimeria* spp. counts under low *vs*. high GIN infection levels. In total, three such comparisons were made [See Additional file 2, Table S2], with significance assessed through permutation.

Our choice of this two-trait, context-dependent framework, rather than others like a continuous reaction norm approach, was based on both biological reasoning and data structure. This will be addressed in more detail in the Discussion section.

## Results

### Prevalence of coinfection and its effect on phenotypic resistance

**Fig. 1:**
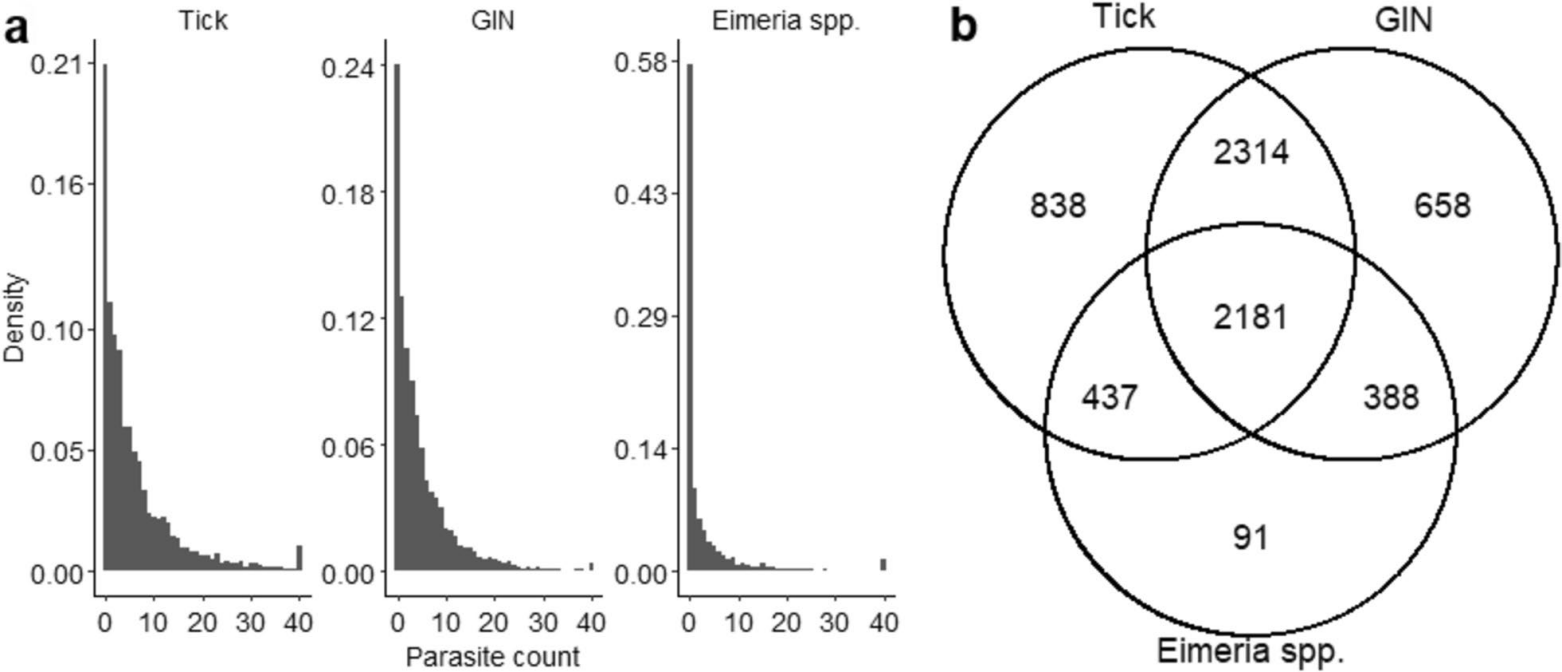
Distributions of tick, GIN and *Eimeria* spp. counts in Nellore calves. **A** Smoothed density plots of observed parasite counts. All raw counts > 40 eggs/oocysts were combined into a single category on the x-axis (73 tick counts > 40, 26 GIN counts > 40, 95 *Eimeria *counts > 40). The true maximum counts in the full dataset were 131 for ticks, 80 for GIN, and 328 for EIM. Means, standard deviations, and medians (raw data) are (mean = 6.38, sd = 8.58, median = 3) for tick, (mean = 4.95, sd = 6.71, median = 3) for GIN, (mean = 3.74, sd = 12.80, median = 0) for EIM. **B** Venn diagram showing numbers of animals positive for one, two, or all three parasite taxa

There was considerable variation in parasite counts among Nellore calves, with right-skewed distributions for all three parasite species considered in this study (Fig. 1a). Simultaneous infections with multiple parasites were found in 5320 (73%) out of 7294 samples, indicating that coinfection is a common phenomenon. Only 387 samples did not contain any of the three parasites (Fig. 1b).

Based on the DIC statistics, accounting for coinfecting parasites (GIN or EIM) in the GLMMs of tick counts did not improve the model fit, and vice versa (Table 1). However, the inclusion of EIM as a coinfecting parasite (either as a class effect or linear covariate) improved the model fit for GIN counts, indicating that coinfection with EIM affects resistance to GIN at the phenotypic level. Specifically, it corresponded to higher GIN counts (posterior mean of linear effect ± standard deviation of 0.0073 ± 0.0012). Conversely, the inclusion of GIN as a coinfecting parasite improved the model fit for EIM, corresponding to higher counts (0.0303 ± 0.0036). This reciprocal association suggests a phenotypic interplay between GIN and EIM. Although the magnitude of these linear effects seems to be small, it is important to remember that parasite counts were not transformed or multiplied by any constant to account for the dilution used in the parasitological examination.

**Table 1:** Posterior means (standard deviations) of additive genetic (${\sigma }_{{a}_{\omega }}^{2}$), permanent environment (${\sigma }_{{c}_{\omega }}^{2}$), and group (${\sigma }_{{g}_{\omega }}^{2}$) variances and deviance information criterion (DIC) from different models for tick (T), GIN (G) and *Eimeria* spp. (E) counts in Nellore calves

Trait	Coinfecting^1^	${\sigma }_{{a}_{\omega }}^{2}$	${\sigma }_{{c}_{\omega }}^{2}$	${\sigma }_{{g}_{\omega }}^{2}$	DIC
T	none	0.156 (0.032)	0.162 (0.024)	1.165 (0.119)	36,474.74
	G (C)	0.156 (0.031)	0.162 (0.024)	1.164 (0.117)	36,477.92
	G (L)	0.157 (0.032)	0.161 (0.024)	1.167 (0.118)	36,477.07
	E (C)	0.157 (0.031)	0.161 (0.024)	1.166 (0.116)	36,477.25
	E (L)	0.157 (0.031)	0.161 (0.025)	1.166 (0.117)	36,476.69
G	none	0.286 (0.049)	0.156 (0.032)	0.551 (0.059)	35,556.39
	T (C)	0.285 (0.048)	0.156 (0.031)	0.551 (0.060)	35,564.36
	T (L)	0.286 (0.049)	0.156 (0.032)	0.551 (0.059)	35,556.69
	E (C)	0.285 (0.049)	0.156 (0.032)	0.551 (0.060)	35,510.76
	E (L)	0.286 (0.048)	0.156 (0.032)	0.551 (0.059)	35,518.56
E	none	0.219 (0.055)	0.247 (0.056)	4.845 (0.536)	23,589.90
	T (C)	0.220 (0.055)	0.247 (0.054)	4.844 (0.541)	23,593.74
	T (L)	0.220 (0.055)	0.247 (0.055)	4.835 (0.535)	23,591.49
	G (C)	0.220 (0.056)	0.246 (0.055)	4.841 (0.539)	23,546.93
	G (L)	0.219 (0.055)	0.246 (0.055)	4.833 (0.539)	23,511.30

^1^Effect of coinfecting parasites as a class effect (C), with low or high levels of coinfection, or as a linear covariable (L)

### Effect of coinfection on genetic and nongenetic variance components for host resistance

**Fig. 2:**
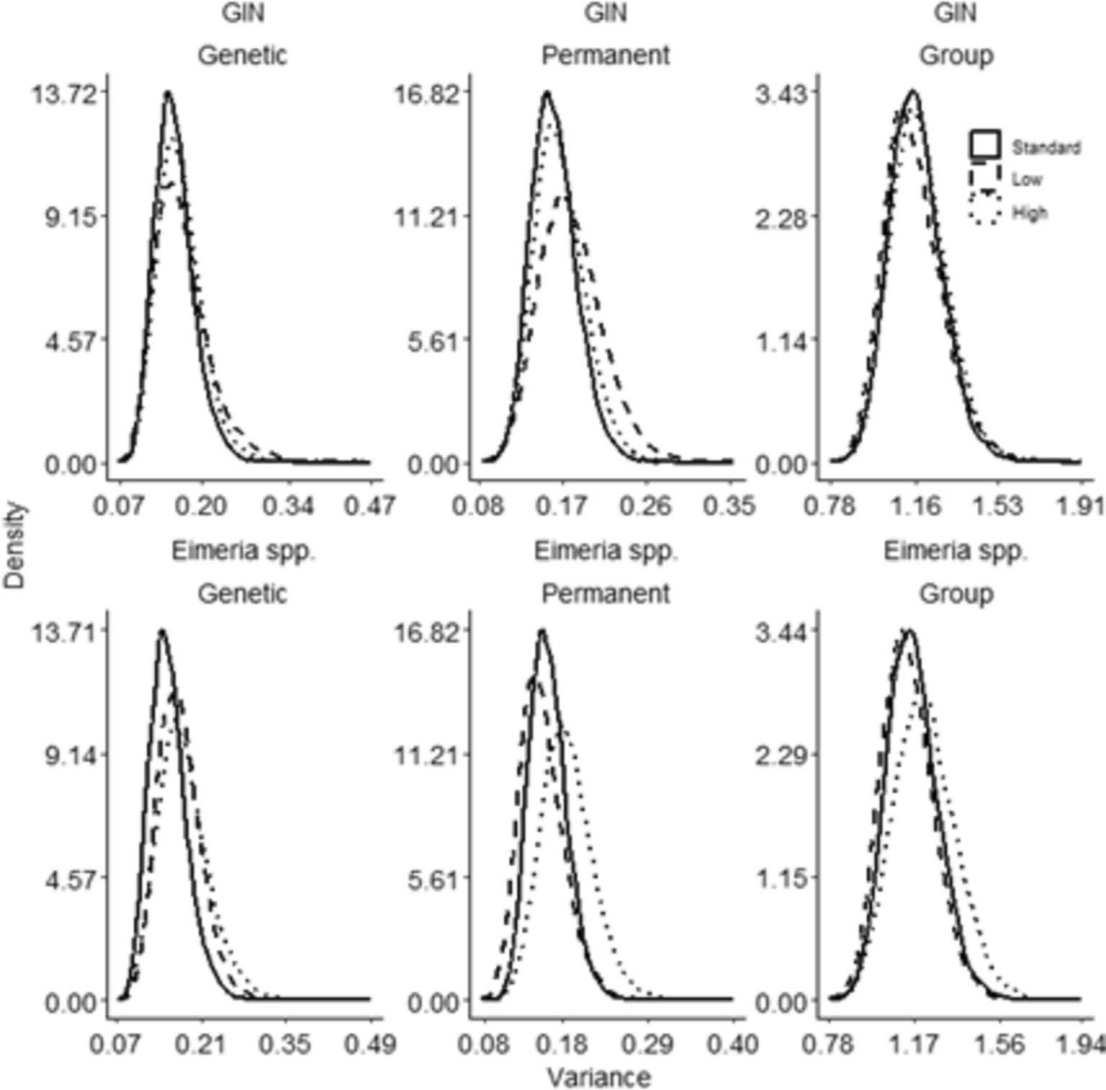
Posterior marginal density distributions of genetic, permanent environmental and group variances for tick counts in Nellore calves under low or high levels of coinfection. Coinfection with GIN (top row) or *Eimeria* spp. (bottom row). The solid lines represent distributions of variance in the standard single trait analysis (full data set of tick counts, without considering coinfection). The dashed and dotted lines represent distributions of variance for tick counts in calves with low or high levels of coinfection, respectively (two-trait analysis)

Despite the observed phenotypic effects of coinfection on GIN and EIM resistance, the inclusion of coinfecting effects in the analyses of resistance to parasites had a negligible effect on the posterior mean estimates of additive genetic, permanent environment and group variances (Table 1). Additionally, the posterior distributions of additive genetic, permanent environment and group variances for ticks and GIN under low or high levels of coinfection with *Eimeria* spp. paralleled those obtained in standard single trait analyses (Fig. 2). The permutation approach revealed that the observed minor deviations in the posterior distributions fell within the range expected from random splitting of ectoparasite counts into two separate data sets [See Additional file 3, Figure S1], suggesting that they should be attributed to sampling effects rather than indicating genuine effects of coinfection. Similarly, the posterior distributions for *Eimeria* spp. also remained consistent with those from standard analyses, ignoring coinfection [See Additional file 3, Figure S2 and S3].

**Fig. 3:**
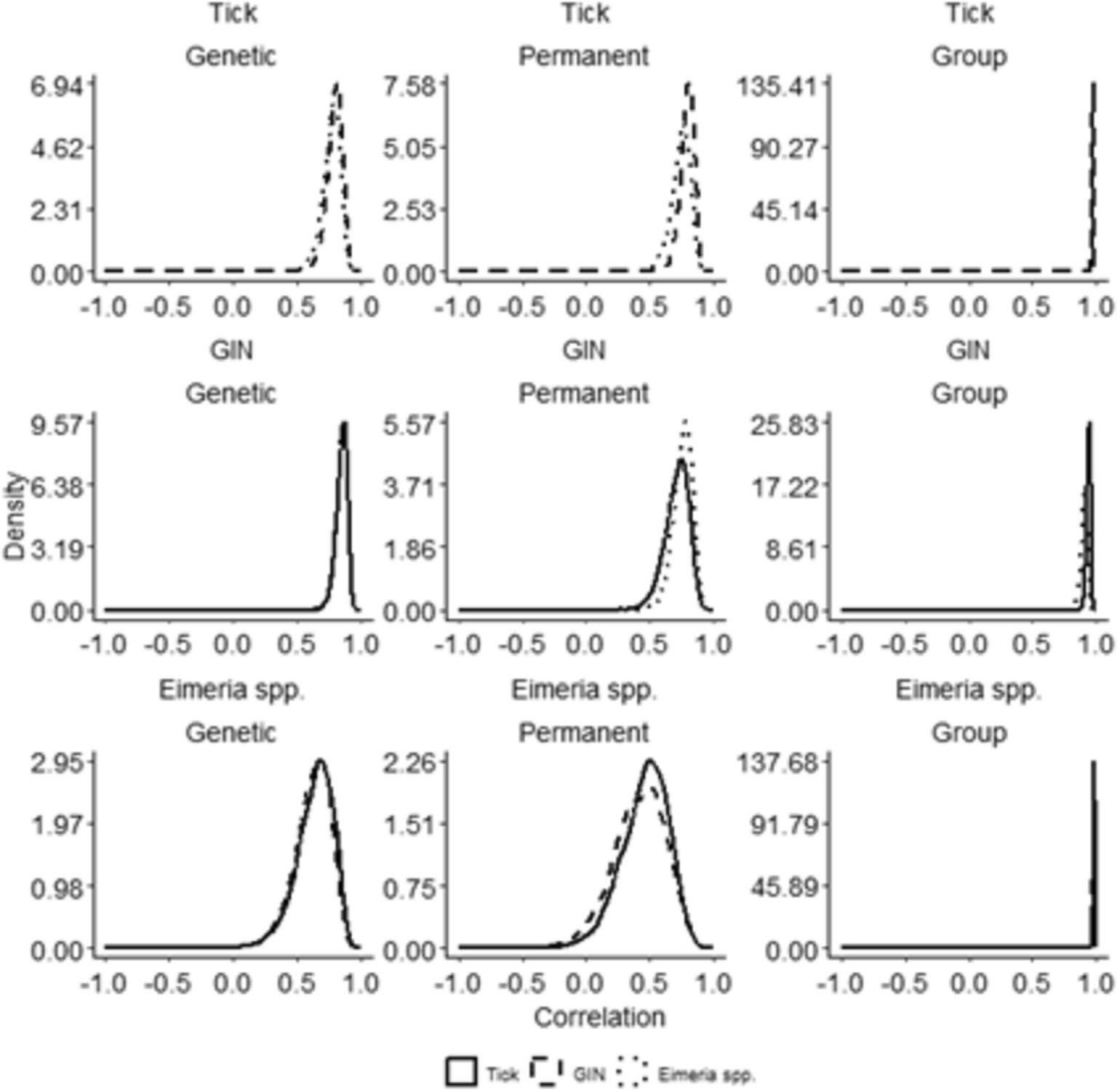
Posterior marginal density distributions of genetic, permanent environmental and group correlations for each parasite. Row 1: Posterior density of the correlation between tick counts in low *vs*. high GIN + EIM coinfection; Row 2: Posterior density of the correlation between GIN counts in low *vs*. high tick + EIM coinfection; Row 3: Posterior density of the correlation between EIM counts in low *vs*. high tick + GIN coinfection. Line styles (solid, dashed, dotted) indicate which secondary parasite defines the coinfection contrast

Genetic correlations between parasite counts under high and low coinfection levels were generally strong, suggesting that host genetic resistance is largely consistent across coinfection contexts. For tick counts, the genetic correlations across coinfection levels with GIN and EIM were 0.80 ± 0.06 and 0.78 ± 0.06, respectively. In the case of GIN, genetic correlations across coinfection with ticks or EIM were both estimated at 0.85 ± 0.04. By contrast, EIM exhibited moderate genetic correlations across coinfection levels: 0.64 ± 0.15 for coinfection with ticks and 0.63 ± 0.14 for coinfection with GIN, suggesting that genetic resistance to EIM may be more sensitive to coinfecting parasite pressures (See Fig. 3 and Additional file 2, Table S3).

Permanent environmental correlations, reflecting consistent individual-level non-genetic effects (e.g., early-life environment or repeated exposure patterns), were of similar magnitude to the genetic correlations across all parasite types. This indicates that individual-specific factors influencing parasite burden tend to persist regardless of coinfection level. Group-level correlations that capture shared environmental influences were consistently high, with posterior means exceeding 0.92 across all comparisons. It is worth mentioning that in Fig. 3 the posterior density for the group‐effect correlations appears flat compared to those for the additive‐genetic and permanent environmental correlations. This strongly suggests that the correlation for the group effect is one. Additionally, we have overlaid all three posteriors on a common x–axis range for direct comparison, which compresses the appearance of the posterior distributions. Thus, the flattened shape does not indicate failure of estimation but simply reflects a high correlation at the parameter boundary. These results highlight the substantial role of shared environmental conditions in shaping parasite burdens, irrespective of the host’s coinfection status.

To assess whether the observed deviations from unity in the genetic correlations truly reflect coinfection-modulated genetic resistance, we applied a permutation-based validation. Across 10 replicated datasets generated by random sampling [see Additional file 3, Fig. S4], the posterior marginal distributions of the correlations closely matched the estimates shown in Fig. 3. This analysis supports the interpretation that the observed deviations from perfect genetic correlation are most likely attributable to sampling noise rather than to a true shift in genetic resistance across coinfection levels.

The posterior marginal distributions of additive genetic, permanent environmental and group variances for the diverse parasite counts obtained from the multiple-trait models (accounting for high and low levels of coinfection with a second parasite) were equivalent to those derived from the single-trait models [See Additional file 3, Figures S5–S7]. Furthermore, the permutation test confirmed that disparities in the posterior distributions associated with different levels of coinfection were within the range of disparities associated with different sets of random samples [See Additional file 3, Figure S8].

Taken together, these results suggest that coinfection has no significant effect on additive genetic, permanent environmental or group variances for host resistance to the different parasite species considered in this study.

### Effect of coinfection on genetic correlations between host resistance to different parasite species

**Fig. 4:**
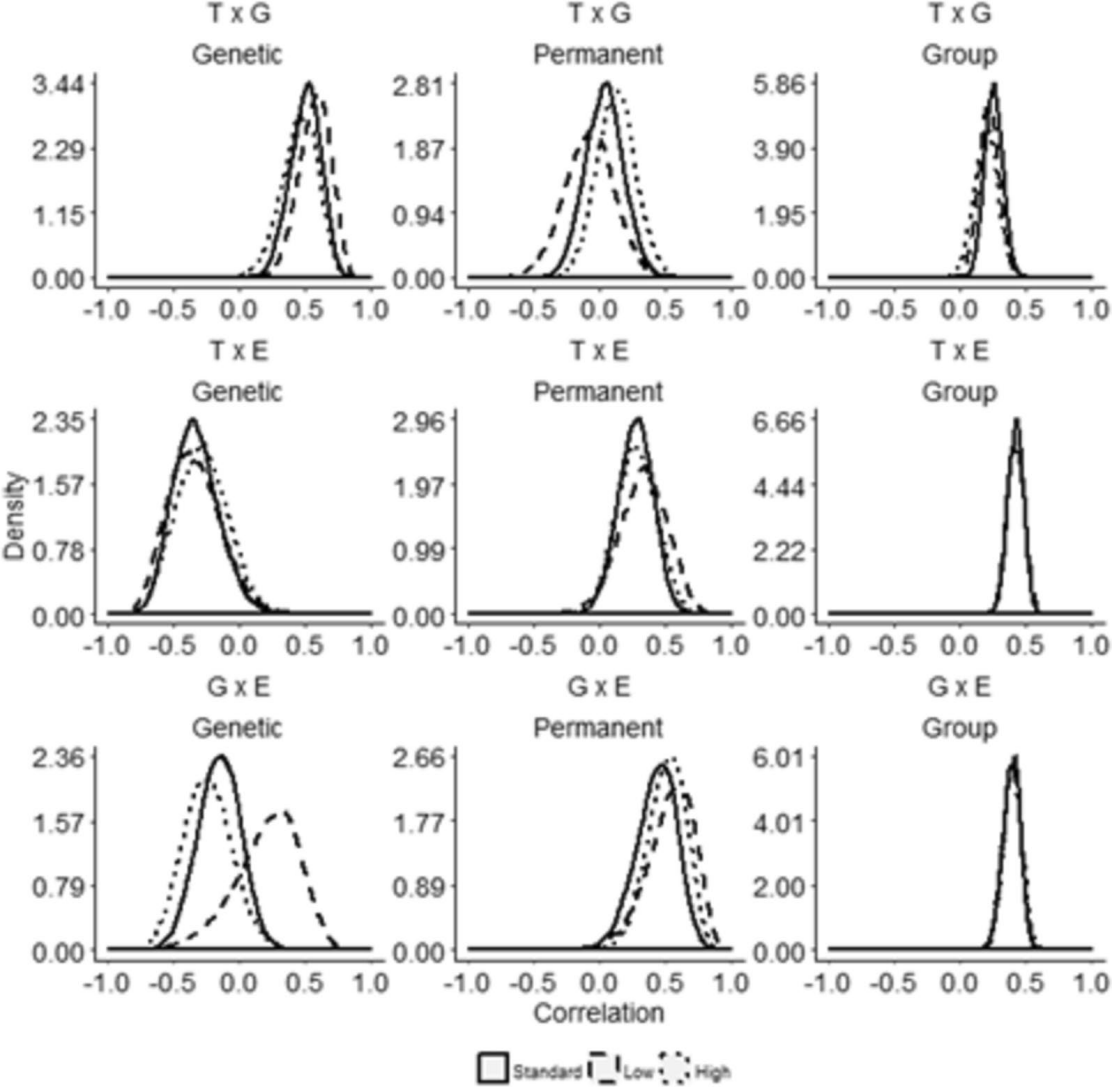
Posterior marginal density distributions of genetic, permanent environmental and group correlations accounting for coinfection. Top row: standard (no coinfection); Middle row: low level of the third parasite’s coinfection; Bottom row: high level of the third parasite’s coinfection. Tick and GIN (T × G, top row), tick and *Eimeria* spp. (T × E, middle row) and GIN and *Eimeria* spp. (G × E, bottom row) counts in yearling Nellore calves with low and high coinfection levels of *Eimeria* spp., GIN, or ticks, respectively. The solid lines represent distributions of correlations in the standard two-parasite trait analysis (full data set of T and G counts, T and E counts, and G and E counts, without considering coinfection). The dotted and dashed lines represent distributions of correlations in the calves with low and high levels of coinfection, respectively

The multiple-trait standard analyses (*i.e.*, not accounting for coinfection) revealed genetic associations between parasite counts, suggesting that host resistance to different parasites is partially shared. Specifically, genetic resistance to ticks was positively correlated with resistance to GIN (0.50 ± 0.12) and negatively correlated with resistance to EIM (− 0.33 ± 0.17). In contrast, the genetic correlation between resistance to GIN and EIM was weak (− 0.16 ± 0.16) and centered near zero, suggesting little to no meaningful association between these traits. This conclusion is supported by the posterior distributions shown on Fig. 4.

Notably, these genetic associations were not substantially altered by coinfection status. Although, the posterior distribution of the GIN–EIM genetic correlation under low coinfection appeared slightly shifted relative to other groups, the variation was within the range observed in 10 random permutation tests [See Additional file 3, Figure S9]. This indicates that the apparent shift is unlikely to result from biological differences in coinfection levels.

For permanent environmental effects, correlations between tick and GIN counts largely overlapped zero, whereas correlations involving *Eimeria* spp. were low to moderately positive, regardless of coinfection status. Similarly, group-level correlations (reflecting shared environmental influences such as management or birth year-season) were consistently positive and ranged from low to moderate across all parasite combinations (Fig. 4).

## Discussion

### Coinfection patterns and phenotypic effects

Our study confirmed that coinfection is a natural occurrence in beef cattle, wherein ticks, GIN and *Eimeria* spp. commonly co-occur. These species are widespread in tropical and subtropical regions, particularly in grazing systems such as those in Brazil [1, 25, 27–29, 31]. Their presence is associated with reduced health, productivity losses, increased carbon footprint, and animal welfare concerns [1, 2, 9, 37].

We observed that phenotypic resistance can be influenced by the combination of coinfecting species. Specifically, GIN and *Eimeria* spp. displayed a reciprocal negative association at the phenotypic level, aligning with previous findings in rodents [38], where coinfection suppressed immunity and increased worm burdens, and in buffaloes [12], where positive associations were found between nematode and coccidia infections in terms of both likelihood and intensity. This interaction may stem from immune modulations [12]. For instance, coinfection led to reductions in nematode-specific IgG and fecal IgA, as well as delayed worm expulsion and prolonged egg shedding in nematodes and lower peak of *Eimeria* spp. oocysts burden [38]. Moreover, *Eimeria* spp. actively reshape the intestinal microenvironment and host immunity in ways that could exacerbate concurrent nematode infection. Weng et al. [9] review how Eimeria‐secreted microneme and rhoptry proteins trigger nuclear factor kappa-B (NF*-κ*B) signaling, leading to elevated IL-10 and transforming growth factor*-β (*TGF*-β*), macrophage polarization, and disruption of tight‐junction integrity, all of which diminish protective Th1/Th17 responses and impair mucosal barriers, creating a permissive niche for other parasites. Similarly, GIN infections are also known to compromise immunity and increase susceptibility to other parasites in cattle, with young animals being particularly vulnerable [6, 39].

In contrast, tick counts were phenotypically unaffected by endoparasitic infections and vice versa. Our findings agree with previous work by Prayaga and Henshall [40], who reported no significant phenotypic correlation between tick burden and worm fecal egg counts in tropical cattle. Ticks primarily provoke a local cutaneous response, where recognition of salivary antigens at the bite site recruits neutrophils and mast cells, drives histamine release, and often elicits a Th1-biased cytokine milieu (*e.g*. IFN-γ, TNF-α) aimed at limiting parasite feeding and attachment [41]. In contrast, gastrointestinal nematodes and *Eimeria* spp. invoke a systemic Th2-dominated response, characterized by IL-4, IL-5 and IL-13 secretion, eosinophilia, mast cell hyperplasia, and mucosal IgA production, which together mediate parasite expulsion, mucosal repair, and regulation of inflammation [42]. These divergent immunological strategies may explain why we observe no phenotypic correlation between tick and GIN/*Eimeria* spp. burdens but a reciprocal association among the endoparasites that share the gut niche. We also hypothesize that given the distinct ecological niches of these parasites (skin vs gut) direct resource competition is unlikely. This spatial separation also helps to explain the absence of observable phenotypic associations. However, studies addressing ecological interactions between external and internal parasites under natural conditions remain scarce.

### Genetic architecture remains stable despite coinfection

Despite the phenotypic interactions observed, our genetic analyses indicated that host genetic resistance to ticks, GIN, and *Eimeria* spp. remained unaffected by coinfection. Estimates of genetic variance and covariance, as well as permanent environmental and group effects, were consistent across infection contexts. This genetic robustness is critical for breeding programs, suggesting that resistance phenotypes recorded under field conditions remain valid and reliable for selection purposes. In practice, this means that genetic gains from selection should not be compromised by coinfection-induced phenotypic noise. Our results support prior studies showing that resistance to these parasites is heritable in Nellore cattle [22, 24–26, 43], reinforcing the feasibility of long-term genetic selection as a sustainable control strategy. The lack of genotype-by-coinfection interactions in variance components underscores the stability of these traits across environmental complexities.

### Genetic and non-genetic correlations between parasites resistance

Phenotypically, resistance to ticks was uncorrelated with resistance to endoparasites. However, we identified significant genetic correlations between these traits, suggesting underlying shared genetic control. Similar results were reported by Prayaga and Henshall [40] and Burrow [44] in cattle populations. These discrepancies may arise from trade-offs between additive genetic and non-additive/environmental effects. Environmental sensitivity of ticks to short-term climatic variation such as humidity and temperature combined with their distinct life cycles may mask phenotypic associations [24]. In contrast, genetic correlations may reflect pleiotropy or linkage between causal loci [45]. For example, genes such as *MAP3K1* have been associated with resistance to both ecto- and endoparasites, and certain single nucleotide polymorphism markers have shown joint effects on resistance to all three parasite species [11].

Our findings suggest that selection for increased tick resistance may also enhance resistance to GIN, but could negatively impact resistance to *Eimeria* spp. Therefore, multiple trait selection strategies (*e.g.*, selection indices or independent culling levels) should be considered to balance potential trade-offs. Permanent environmental correlations, which encompass both non-additive genetic and environmental influences (*e.g.*, early-life immune priming), were moderately strong between EIM and both ticks and GIN. According to classical quantitative genetics theory, the phenotypic correlation between two traits can be partitioned into coheritability [46], the proportion explained by shared genetic effects, and coenvironmentability [47], which captures the contribution of shared environmental factors. This framework helps clarify situations where environmental correlations act in the opposite direction of genetic correlations, as observed in our results, ultimately diluting the phenotypic association between traits despite genetic antagonism or synergy.

Group effects, reflecting shared management conditions (*e.g.,* season, year, location), explained substantial variation in parasite burden. Notably, group‐effect correlations across multiple parasite traits were consistently high (posterior means > 0.92), suggesting that similar environmental factors shaped the expression of resistance across different parasite species. The highest parasite counts observed during the rainy season are consistent with previous studies and the known seasonal dynamics in Brazil [11, 12, 48, 49]. Altogether, these findings support the interpretation that consistent environmental pressures contribute significantly to the phenotypic expression of resistance traits.

### Methodological considerations and limitations

To prevent confounding due to sampling artifacts, we employed a permutation-based validation approach to test coinfection effects on genetic parameters. This step was essential to differentiate true biological effects from spurious variation that could mimic genotype-by-environment interactions. While we modeled infection context discretely, future work may benefit from using reaction norm models to assess coinfection as a continuous or threshold trait, especially in larger datasets. However, it is important to recognize that coinfection as an environmental covariate should be considered with caution because coinfection represents biological interactions between host immune responses and parasite competition, which may not conform to linear assumptions.

Our results also emphasize that the phenotypic impact of coinfection is context dependent. As shown in African buffalo, coinfection outcomes vary with host age, sex, and season [12]. These demographic and ecological variables can alter infection intensity and parasite aggregation, reinforcing the need for cautious interpretation of coinfection effects across systems. Finally, we acknowledge that ticks were not examined for pathogens such as *Babesia* or *Anaplasma,* nor were specific diagnostics performed to rule out coinfections with other parasites (*e.g.*, trematodes); incorporating such screening represents an important avenue for future research. Such pathogen screening could uncover additional layers of host–pathogen dynamics, clarify the role of vector‐borne agents in shaping genetic resistance, and ultimately inform breeding strategies in a broader spectrum.

### Implications for breeding and management strategies

Our study offers a rare and comprehensive analysis of coinfection's impact on resistance traits over a 7-year longitudinal dataset. While previous literature suggests that coinfection can influence treatment outcomes and parasite burdens [11, 24–26], few studies have assessed its influence on the genetic architecture of resistance. Importantly, our findings demonstrate that genetic selection for increased resistance to individual parasites does not adversely affect resistance to others. For example, selecting for increased resistance to ticks improves GIN resistance, but does not impact *Eimeria* spp. resistance. Likewise, selection for GIN resistance appears neutral with respect to the other parasites. These results support the potential of breeding programs to enhance resistance without exacerbating susceptibility to coexisting parasites.

Although this study did not directly evaluate selection against coinfection, our results imply that improving resistance to individual parasites would indirectly reduce the likelihood of coinfection. Future work should explore this further, especially at the molecular level. Studies identifying genomic regions such as *MAP3K1, IL-13,* or *TLRs* may uncover the immunogenetic basis of resistance under coinfection [11, 50, 51].

In summary, while coinfection clearly modulates phenotypic resistance and may affect host condition, it does not alter the additive genetic control of resistance traits in Nellore cattle. Thus, genetic selection remains a stable and effective strategy to mitigate parasite burdens in tropical grazing systems, even within complex coinfection scenarios. Future studies should delve deeper into the immunological and genomic mechanisms of coinfection to refine selection targets and complement sustainable parasite control strategies.

## Conclusions

Our study provides the first comprehensive evaluation of how coinfection influences genetic resistance to multiple parasites in cattle under natural grazing conditions. While coinfection was highly prevalent and showed species-specific phenotypic interactions, we found no evidence that it alters genetic variances, covariances, or correlations for resistance to ticks, GIN, or *Eimeria* spp. These results demonstrate that genetic resistance to each parasite is stable and robust across different coinfection contexts. Importantly, favorable or antagonistic genetic correlations between parasite resistances were unaffected by coinfection, suggesting that genetic selection strategies targeting single or multiple parasites remain valid under natural coinfection pressure. Therefore, breeders can confidently apply multiple-trait selection methods to enhance host resistance without concern for hidden genotype-by-coinfection interactions. Although further research is needed to investigate the genetic basis of coinfection itself and immune-related gene interactions, our findings reinforce the viability of genetic selection as a sustainable tool in integrated parasite control programs.

## Supplementary Information

Additional file 1. **Data Analysis.** Data analysis comparing Poisson and Negative Binomial animal models and their zero-inflated versions.

Additional file 2. **Table S1.** Descriptive statistics of tick (T), GIN (G) and *Eimeria* spp. (E) counts in yearling Nellore calves with low and high levels of coinfection for single trait analyses. **Table S2.** Descriptive statistics of tick (T), GIN (G) and *Eimeria* spp. (E) counts in yearling Nellore calves with low and high levels of coinfection for multiple trait analyses. **Table S3.** Posterior summaries of correlations between coinfection levels for each parasite across variance components

Additional file 3. **Figure S1.** Posterior distributions of genetic, permanent environmental and group variances for tick (top row), GIN (middle row) and *Eimeria spp*. (bottom row) counts in yearling Nellore calves. The black solid lines represent distributions of variance in the standard single-trait analysis. The gray (solid or dashed) lines represent distributions of variances in the two randomly disjoint groups containing approximately 50% of the records in each group (two-trait analysis, 10 replicates). **Figure S2.** Posterior distributions of genetic, group and permanent environmental variances for GIN counts in yearling Nellore calves under low or high levels of coinfection. Coinfection with ticks (top row) or *Eimeria* spp. (bottom row). The solid lines represent distributions of variance in the standard single-trait analysis (all GIN were counted together, without considering coinfection). The dashed and dotted lines represent distributions of variance for GIN counts in calves with low or high levels of coinfection, respectively (two-trait analysis). **Figure S3.** Posterior distributions of genetic, group and permanent environmental variances for *Eimeria* spp. counts in yearling Nellore calves under low or high levels of coinfection. Coinfection with ticks (top row) or GIN (bottom row). The solid lines represent distributions of variance in the standard single trait analysis (all Eimeria spp. were counted together, without considering coinfection). The dashed and dotted lines represent distributions of variances for Eimeria spp. counts in calves with low or high levels of coinfection, respectively (two-trait analysis). **Figure S4.** Posterior distributions of genetic, group and permanent environmental correlations among parasites. Tick (top row), GIN (middle row), and *Eimeria* spp*.* (bottom row) counts in yearling Nellore calves in two randomly disjointed groups containing approximately 50% records each (10 replicates). **Figure S5.** Posterior distributions of genetic, group and permanent environmental variances for tick counts in yearling Nellore calves from two-parasite trait analysis. Coinfection with GIN (top row) or with *Eimeria* spp. (bottom row). Solid lines represent distributions of variance in the standard two-trait analysis (all tick and GIN counts together and all tick and *Eimeria* spp. counts together, without considering coinfection). Dashed and dotted lines represent distributions of variances for tick counts under low or high levels of coinfection with *Eimeria* spp. (top row) or with GIN (bottom row). **Figure S6.** Posterior distributions of genetic, group and permanent environmental variances for GIN counts in yearling Nellore calves from two-parasite trait analysis. Coinfection with ticks (top row) or with *Eimeria* spp. (bottom row). Solid lines represent distributions of variance in the standard two-trait analysis (all GIN and tick counts together and all GIN and *Eimeria* spp. counts together, without considering coinfection). Dashed and dotted lines represent distributions of variances for GIN counts under low or high levels of coinfection with *Eimeria* spp. (top row) or with ticks (bottom row). **Figure S7.** Posterior distributions of genetic, group and permanent environmental variances for *Eimeria* spp. counts in yearling Nellore calves, from two-parasite trait analysis. Coinfection with ticks (top row) or with GIN (bottom row). Solid lines represent distributions of variance in the standard two-trait analysis (all *Eimeria* spp. and tick counts together and all *Eimeria* spp. and GIN counts together, without considering coinfection). The dashed and dotted lines represent distributions of variances for *Eimeria* spp. counts under low or high levels of coinfection with ticks (top row) or with GIN (bottom row). **Figure S8.** Posterior distributions of genetic, permanent and group variances for tick (top row), GIN (middle row) and *Eimeria* spp. (bottom row) counts in yearling Nellore calves from two-trait analyses. Black lines represent distributions from complete data sets and single trait analysis. The gray lines represent distributions from 10 replicates of random sampling of approximately 50% of the data. The solid or dashed lines represent the two sets of analyses for each trait (tick with GIN or with *Eimeria* spp., GIN with ticks or with *Eimeria* spp., and *Eimeria* spp. with ticks or with GIN). **Figure S9.** Posterior distributions of genetic, group and permanent environmental correlations. Correlations between tick and GIN (T × G, top row), ticks and *Eimeria* spp. (T × E, middle row) and GIN and *Eimeria* spp. (G × E, bottom row) counts in yearling Nellore calves. The black lines represent distributions from complete data sets. The gray lines represent distributions from 10 replicates of random sampling of approximately 50% of the data.

## Data Availability

The datasets used during the current study are available from the corresponding author on reasonable request.
