# Supplementary material for: Coinfection affects the phenotypic but not genetic resistance of cattle to common parasites
Source: Genet Sel Evol. 2025 Oct 7;57:55. doi: 10.1186/s12711-025-01003-y (PMC12506400; doi:10.1186/s12711-025-01003-y)
Supplement: Supplementary file 1 — Additional file 1. Data Analysis. Data analysis comparing Poisson and Negative Binomial animal models and their zero-inflated versions. [file 12711_2025_1003_MOESM1_ESM.docx]

**Additional file 1, Data Analysis**

**Data Analysis comparing Poisson and Negative Binomial animal models and their zero-inflated versions**

The data were analyzed via Poisson (P) and negative binomial (NB) animal models and their zero-inflated versions (ZIP and ZINB, respectively). Zero-inflated models assume that the data are generated from a mixture of two distributions. For these mixture models, the probability mass function of the response variable $Y_{i}$ (parasite count) is as follows:

$Pr\left( Y_{ij}=y_{ij} | \eta,\theta_{i} \right)=\left\{ \begin{matrix} \eta+f\left( Y_{ij}=0 | \theta_{i} \right)\left( 1-\eta\right), y_{ij}=0 & \\ f\left( Y_{i}=y_{ij} | \theta_{i} \right)\left( 1-\eta\right), y_{ij}=0, 1,2,\ldots& 0\leq\eta\leq1 \end{matrix} \right.$,

where $Y_{i}$ has a probability mass function corresponding to a Poisson or negative binomial distribution defined by a set of parameters $\theta_{i}$, which is assigned a probability $\left( 1-\eta\right)$ and a degenerate distribution supported at zero with probability $\eta$. Furthermore, the standard or nonzero-inflated version of the models is generated by setting $\eta=0$.

For the Poisson model $\theta_{i}=\left( \lambda_{i} \right)$, the probability mass function is as follows:

$f\left( Y_{i}=y_{i} | \lambda_{i} \right)=\frac{\lambda_{i}^{y_{i}}exp\left( -\lambda_{i} \right)}{y_{i}!}, \lambda_{i}>0, Y_{i}=0, 1, \ldots$,

with mean and variance $E\left( Y_{i} | \lambda_{i} \right)=Var\left( Y_{i} | \lambda_{i} \right)=\lambda_{i}$.

For the negative binomial model $\theta=\left( \omega_{i}, \kappa\right)$, the probability mass function is as follows:

$f\left( Y_{ij}=y_{ij} | \omega_{i},\kappa\right)=\frac{\Gamma\left( \kappa+y_{ij} \right)}{\Gamma\left( \kappa\right)y_{ij}!}\left( \frac{\omega_{i}}{\kappa+\omega_{i}} \right)^{y_{ij}}\left( \frac{\kappa}{\kappa+\omega_{i}} \right)^{\kappa}$,

where $\omega$ is the mean and where $\kappa$ is the shape parameter, which approaches the Poisson distribution as $\kappa\to\infty$, with $E\left( Y_{ij} | \omega_{i},\kappa\right)=\omega_{i}$ and $Var\left( Y_{ij} | \omega_{i},\kappa\right)=\omega_{i}+\frac{\omega_{i}^{2}}{\kappa}$.

Furthermore, we defined $\ln\lambda=\left\{ \ln\lambda_{i} \right\}_{i=1}^{n}$ and $\ln\omega=\left\{ \ln\omega_{i} \right\}_{i=1}^{n}$ for the Poisson and negative binomial models, respectively. The underlying linear models for $\ln\lambda$ and $\ln\omega$ are as follows:

$\ln\lambda=Xb_{\lambda}+Z_{1}a_{\lambda}+Z_{2}p_{\lambda}+Z_{3}g_{\lambda}$**,**

and

$\ln\omega=Xb_{\omega}+Z_{1}a_{\omega}+Z_{2}p_{\omega}+Z_{3}g_{\omega}$,

where $b_{\lambda}\left( b_{\omega} \right)$ is the age effect. $a_{\lambda}\left( a_{\omega} \right)$, $p_{\lambda}\left( p_{\omega} \right)$, and $g_{\lambda}\left( g_{\omega} \right)$ are the vectors of genetic random effects, permanent environment random effects and group random effects, respectively. $X$, $Z_{1}$, $Z_{2}$ and $Z_{3}$ are the corresponding incidence matrices. The group effect accounts for the year-season of birth, group of management and date of measurement effects.

The vectors of genetic random effects, permanent environment random effects and group random effects are assumed to follow normal distributions:

$a_{\lambda}\left| A,\sigma_{a_{\lambda}}^{2} \right.\sim N\left( 0,A\sigma_{a_{\lambda}}^{2} \right)$, $a_{\omega}\left| A, \right.\sigma_{a_{\omega}}^{2}\sim N\left( 0,A\sigma_{a_{\omega}}^{2} \right)$,

$p_{\lambda}\left| I_{P},\sigma_{p_{\lambda}}^{2} \right.\sim N\left( 0,I_{P}\sigma_{p_{\lambda}}^{2} \right)$, $p_{\omega}\left| I_{P}, \right.\sigma_{p_{\omega}}^{2}\sim N\left( 0,I_{P}\sigma_{p_{\omega}}^{2} \right)$,

$g_{\lambda}\left| I_{G},\sigma_{g_{\lambda}}^{2} \right.\sim N\left( 0,I_{G}\sigma_{g_{\lambda}}^{2} \right)$, $g_{\omega}\left| I_{G}, \right.\sigma_{g_{\omega}}^{2}\sim N\left( 0,I_{G}\sigma_{g_{\omega}}^{2} \right)$,

where $A$ is the additive genetic relationship matrix (with 5,919 animals); $\sigma_{a_{\lambda}}^{2}$ and $\sigma_{a_{\omega}}^{2}$ are additive genetic variances; $I_{P}$ is the identity matrix of order $P$, the number of animals with records (1,712);$\sigma_{p_{\lambda}}^{2}$ and $\sigma_{p_{\omega}}^{2}$ are permanent environment variances; $I_{G}$ is the identity matrix of order $G$, the number of groups (228); and $\sigma_{g_{\lambda}}^{2}$ and $\sigma_{g_{\omega}}^{2}$ are group variances. Prior distributions for the age covariate *b* and variances were the normal and inverse-Gamma distributions, respectively (0.5, 0.5).

The models were compared via the deviance information criterion (DIC). DIC compares the global fit of different models, accounting for their complexities. For each model M, the DIC was computed as:

${DIC}_{M}=2\bar{D}_{M}-D\left( \bar{\theta}_{M} \right)$,

where $\bar{D}_{M}$ is the posterior expectation of the deviance $D\left( \theta_{M} \right)$ and where $D\left( \bar{\theta}_{M} \right)=-2log\left( p\left( y | \theta_{M} \right) \right)$ is the deviance evaluated at the posterior mean estimate of the parameter vector $\theta_{M}$. Models with a smaller DIC exhibit a better global fit, and a difference in DIC of more than 7 units is considered significant.

The DIC results indicated that the NB and ZINB models were better in terms of goodness of fit than the P and ZIP models were (Table SI 1). This is probably because the Poisson distribution assumes equal mean and variance, whereas the negative binomial distribution allows the data to be over dispersed. However, the DIC did not provide circumstantial evidence that the ZINB model better fit the data than the NB model did. Hence, the NB model was used for further analyses.

The posterior means of the zero-inflated factor in the ZINB models (tick: 0.0003, gastrointestinal nematodes: 0.0004, and *Eimeria* spp.: 0.0005) were close to zero. The posterior distributions of genetic, group and permanent environmental effects under the NB and ZINB models were almost the same for the three traits. The posterior distributions of genetic, group and permanent environmental covariances and correlations for every pair of traits under the NB and ZINB models were also similar (data not shown). These results supported the decision of fitting binomial distributions for the data in the present work.

**Table SI 1 Deviance information criterion for models with Poisson (P), negative binomial (NB), zero-inflated P (ZIP) and zero-inflated NB (ZINB) distributions for fitting tick, gastrointestinal nematode and *Eimeria* spp. counts in Nellore calves.**

| **Distribution** | **Tick** | **GIN** | ***Eimeria* spp.** |
| --- | --- | --- | --- |
| P | 40,112.17 | 40,212.86 | 37,596.00 |
| NB | 36,474.74 | 35,556.39 | 23,589.90 |
| ZIP | 39,165.33 | 39,025.41 | 33,375.92 |
| ZINB | 36,449.99 | 35,549.91 | 23,590.08 |
