# Supplementary material for: Coinfection affects the phenotypic but not genetic resistance of cattle to common parasites
Source: Genet Sel Evol. 2025 Oct 7;57:55. doi: 10.1186/s12711-025-01003-y (PMC12506400; doi:10.1186/s12711-025-01003-y)
Supplement: Supplementary file 2 — Additional file 2. Table S1. Descriptive statistics of tick (T), GIN (G) and Eimeria spp. (E) counts in yearling Nellore calves with low and high levels of coinfection for single trait analyses. Table S2. Descriptive statistics of tick (T), GIN (G) and Eimeria spp. (E) counts in yearling Nellore calves with low and high levels of coinfection for multiple trait analyses. Table S3. Posterior summaries of correlations between coinfection levels for each parasite across variance components [file 12711_2025_1003_MOESM2_ESM.docx]

**Additional file 2**

**Table S1 Descriptive statistics of tick (T), GIN (G) and *Eimeria* spp. (E) counts in yearling Nellore calves with low and high levels of coinfection for single trait analyses.**

| **Parasite^1^** | | **Level of coinfection** | | | | | | | |
| --- | --- | --- | --- | --- | --- | --- | --- | --- | --- |
|  |  | **Low** | | | | **High** | | | |
| **Main** | **Coinfecting** | **N** | **Mean** | **SD** | **Range** | **N** | **Mean** | **SD** | **Range** |
| T | G | 3,469 | 5.4 | 7.3 | 0 to 80 | 3,825 | 7.3 | 9.5 | 0 to 131 |
| T | E | 4,197 | 5.3 | 7.9 | 0 to 131 | 3,097 | 7.8 | 9.2 | 0 to 80 |
| G | T | 3,042 | 4.3 | 6.4 | 0 to 80 | 4,252 | 5.4 | 6.9 | 0 to 80 |
| G | E | 4,197 | 4.0 | 5.9 | 0 to 80 | 3,097 | 6.2 | 7.5 | 0 to 80 |
| E | T | 3,042 | 3.0 | 11.9 | 0 to 284 | 4,252 | 4.3 | 13.4 | 0 to 328 |
| E | G | 3,469 | 2.4 | 9.1 | 0 to 213 | 3,825 | 5.0 | 15.3 | 0 to 328 |

^1^Same parasite (main), under different levels of coinfection.

**Table S2 Descriptive statistics of tick (T), GIN (G) and *Eimeria* spp. (E) counts in yearling Nellore calves with low and high levels of coinfection for multiple trait analyses.**

| **Parasite^1^** | | | **Level of coinfection** | **N** | **P1** | | | **P2** | | |
| --- | --- | --- | --- | --- | --- | --- | --- | --- | --- | --- |
| **P1** | **P2** | **P3** |  |  | **Mean** | **SD** | **Range** | **Mean** | **SD** | **Range** |
| T | G | E | Low | 4,197 | 5.3 | 7.9 | 0 to 131 | 4.0 | 5.9 | 0 to 80 |
| T | G | E | High | 3,097 | 7.8 | 9.2 | 0 to 80 | 6.2 | 7.5 | 0 to 80 |
| T | E | G | Low | 3,469 | 5.4 | 7.3 | 0 to 80 | 2.4 | 9.1 | 0 to 213 |
| T | E | G | High | 3,825 | 7.3 | 9.5 | 0 to 131 | 5.0 | 15.3 | 0 to 328 |
| G | E | T | Low | 3,042 | 4.3 | 6.4 | 0 to 80 | 3.0 | 11.9 | 0 to 284 |
| G | E | T | High | 4,252 | 5.4 | 6.9 | 0 to 80 | 4.3 | 13.4 | 0 to 328 |

^1^Different parasites (P1 and P2), under the same level of coinfection with parasite P3.

**Table S3 Posterior summaries of correlations between coinfection levels for tick (T), GIN (G) and Eimeria spp. (E) across variance components.**

| **Parasite** | **VC** | **Coinfecting** | **Mean** | **Median** | **SD** | **95% HPD** |
| --- | --- | --- | --- | --- | --- | --- |
| E | Genetic | G | 0.63 | 0.64 | 0.14 | 0.35 – 0.88 |
| E | Genetic | T | 0.64 | 0.66 | 0.14 | 0.36 – 0.89 |
| E | Group | G | 0.98 | 0.98 | 0.01 | 0.97 – 0.99 |
| E | Group | T | 0.98 | 0.98 | 0.01 | 0.98 – 0.99 |
| E | Permanent | G | 0.41 | 0.43 | 0.20 | 0.03 – 0.80 |
| E | Permanent | T | 0.47 | 0.48 | 0.18 | 0.12 – 0.80 |
| G | Genetic | E | 0.85 | 0.86 | 0.04 | 0.77 – 0.94 |
| G | Genetic | T | 0.85 | 0.86 | 0.04 | 0.77 – 0.94 |
| G | Group | E | 0.90 | 0.91 | 0.03 | 0.86 – 0.96 |
| G | Group | T | 0.94 | 0.94 | 0.02 | 0.91 – 0.97 |
| G | Permanent | E | 0.76 | 0.77 | 0.08 | 0.60 – 0.89 |
| G | Permanent | T | 0.71 | 0.73 | 0.09 | 0.53 – 0.88 |
| T | Genetic | E | 0.78 | 0.78 | 0.06 | 0.64 – 0.90 |
| T | Genetic | G | 0.80 | 0.81 | 0.06 | 0.69 – 0.91 |
| T | Group | E | 0.97 | 0.97 | 0.01 | 0.96 – 0.99 |
| T | Group | G | 0.98 | 0.98 | 0.01 | 0.97 – 0.99 |
| T | Permanent | E | 0.75 | 0.75 | 0.07 | 0.60 – 0.88 |
| T | Permanent | G | 0.80 | 0.80 | 0.05 | 0.70 – 0.90 |

Columns report posterior mean, median, standard deviation (SD), and 95% highest posterior density interval (HPD). “Parasite” = focal parasite trait (Tick, GIN = gastrointestinal nematodes, *Eimeria* spp.); “VC” = variance component (Genetic = additive genetic, Permanent = permanent environmental, Group = shared contemporary group); “coinfecting” = coinfecting parasite defining the two levels. SDs ranged from 0.01 to 0.20 (median = 0.06).
