# Supplementary material for: Coinfection affects the phenotypic but not genetic resistance of cattle to common parasites
Source: Genet Sel Evol. 2025 Oct 7;57:55. doi: 10.1186/s12711-025-01003-y (PMC12506400; doi:10.1186/s12711-025-01003-y)

**Additional file 3**

**Additional file 3, Figure S1**


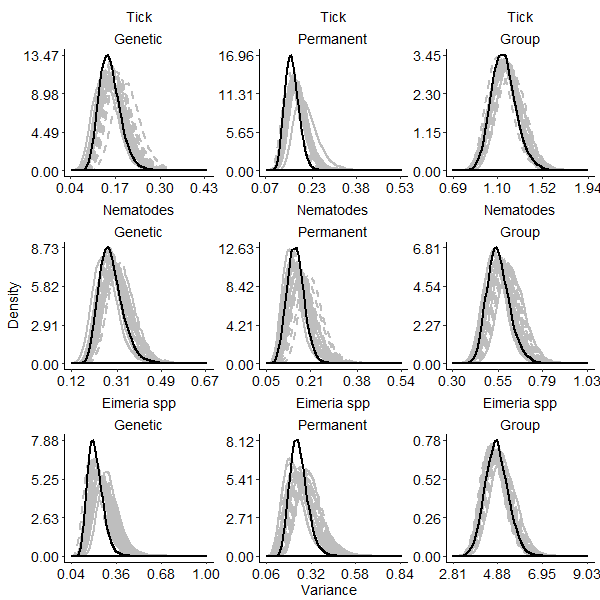


**Additional file 3, Figure S2**


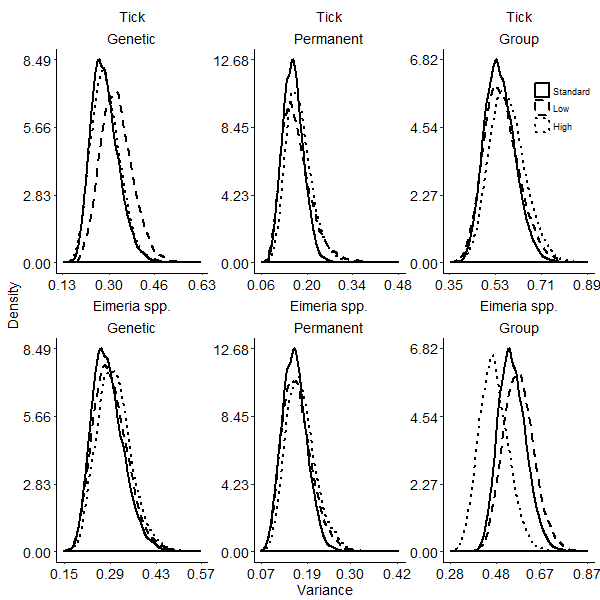


**Additional file 3, Figure S3**


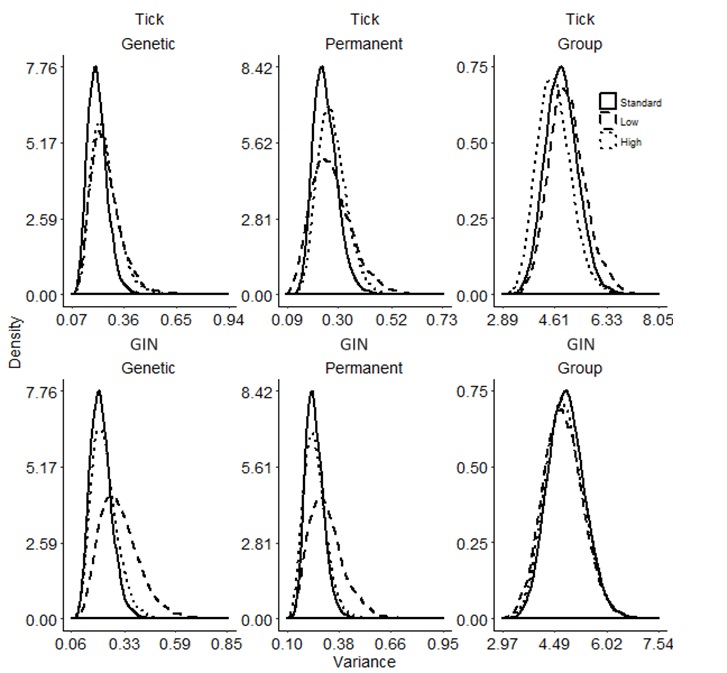


**Additional file 3, Figure S4**


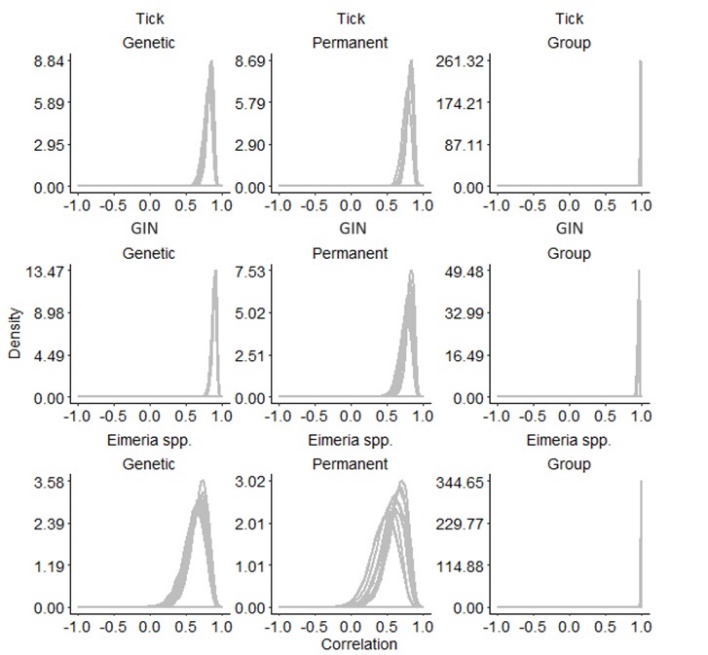


**Additional file 3, Figure S5**


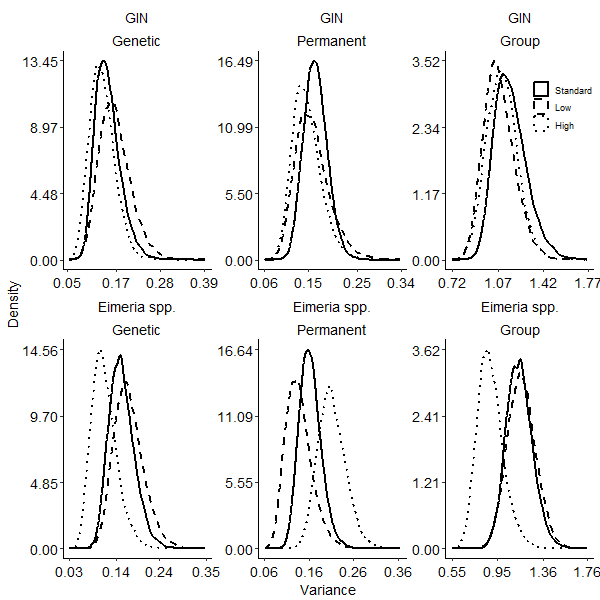


**Additional file 3, Figure S6**


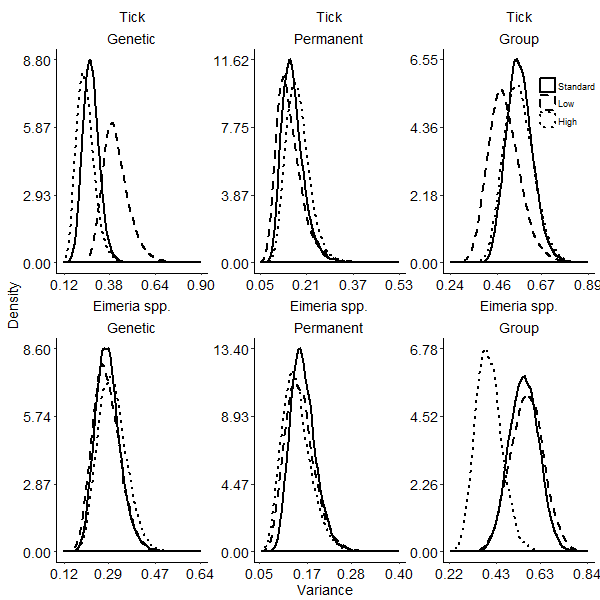


**Additional file 3, Figure S7**


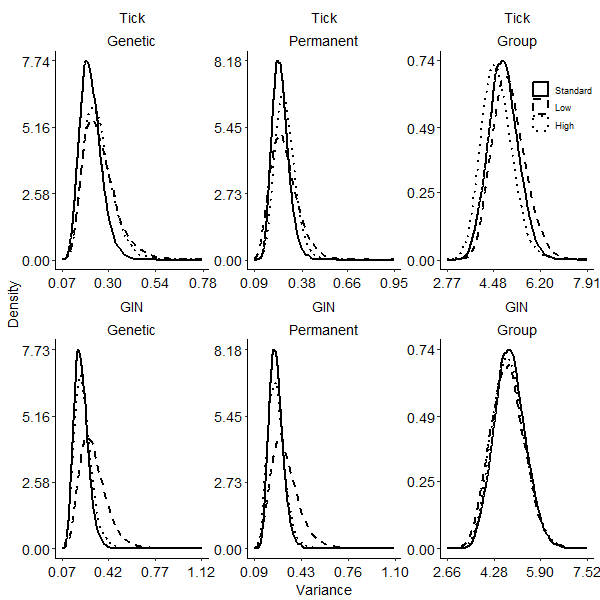


**Additional file 3, Figure S8**


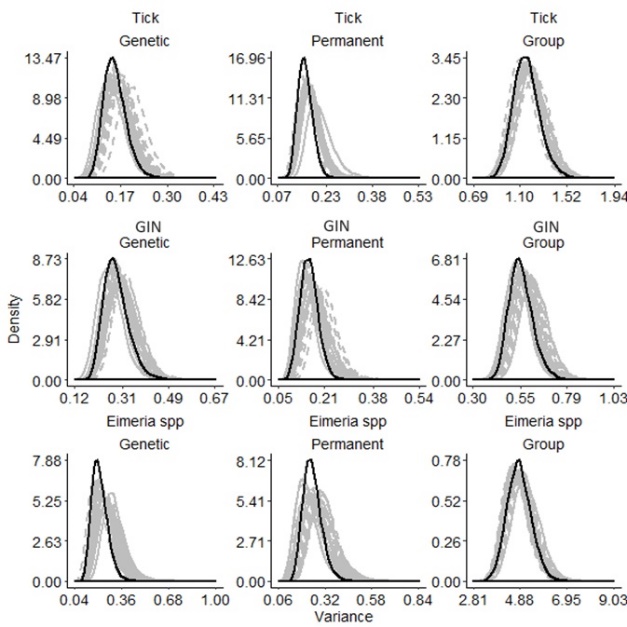


**Additional file 3, Figure S9**


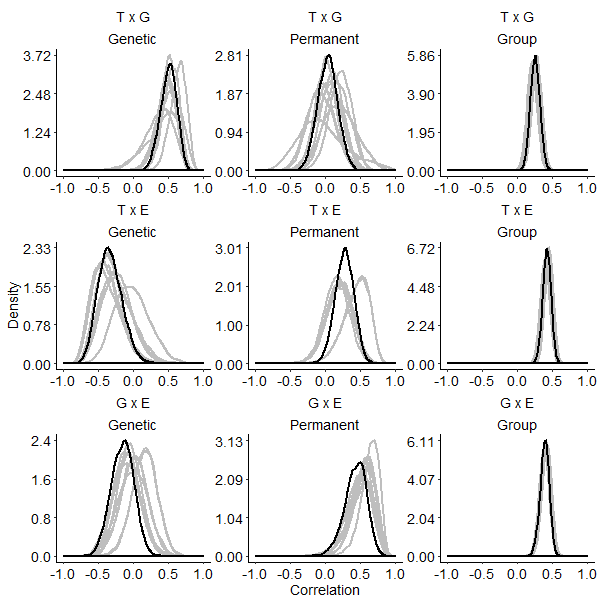

Supplement: Supplementary file 3 — Additional file 3. Figure S1. Posterior distributions of genetic, permanent environmental and group variances for tick (top row), GIN (middle row) and Eimeria spp. (bottom row) counts in yearling Nellore calves. The black solid lines represent distributions of variance in the standard single-trait analysis. The gray (solid or dashed) lines represent distributions of variances in the two randomly disjoint groups containing approximately 50% of the records in each group (two-trait analysis, 10 replicates). Figure S2. Posterior distributions of genetic, group and permanent environmental variances for GIN counts in yearling Nellore calves under low or high levels of coinfection. Coinfection with ticks (top row) or Eimeria spp. (bottom row). The solid lines represent distributions of variance in the standard single-trait analysis (all GIN were counted together, without considering coinfection). The dashed and dotted lines represent distributions of variance for GIN counts in calves with low or high levels of coinfection, respectively (two-trait analysis). Figure S3. Posterior distributions of genetic, group and permanent environmental variances for Eimeria spp. counts in yearling Nellore calves under low or high levels of coinfection. Coinfection with ticks (top row) or GIN (bottom row). The solid lines represent distributions of variance in the standard single trait analysis (all Eimeria spp. were counted together, without considering coinfection). The dashed and dotted lines represent distributions of variances for Eimeria spp. counts in calves with low or high levels of coinfection, respectively (two-trait analysis). Figure S4. Posterior distributions of genetic, group and permanent environmental correlations among parasites. Tick (top row), GIN (middle row), and Eimeria spp. (bottom row) counts in yearling Nellore calves in two randomly disjointed groups containing approximately 50% records each (10 replicates). Figure S5. Posterior distributions of genetic, group an [file 12711_2025_1003_MOESM3_ESM.docx]
